# Supplementary material for: The Gut Bacteria Dysbiosis Contributes to Chronic Graft-Versus-Host Disease Associated With a Treg/Th1 Ratio Imbalance
Source: Front Microbiol. 2022 Sep 8;13:813576. doi: 10.3389/fmicb.2022.813576 (PMC9493085; doi:10.3389/fmicb.2022.813576)
Supplement: Supplementary file 1 [file Data_Sheet_1.zip › P101SC18090073-01-B1-3-4_result/04.BetaDiversity/MetaStat/class/boxplot/figures/p__Actinobacteria_c__unidentified_Actinobacteria.pdf]

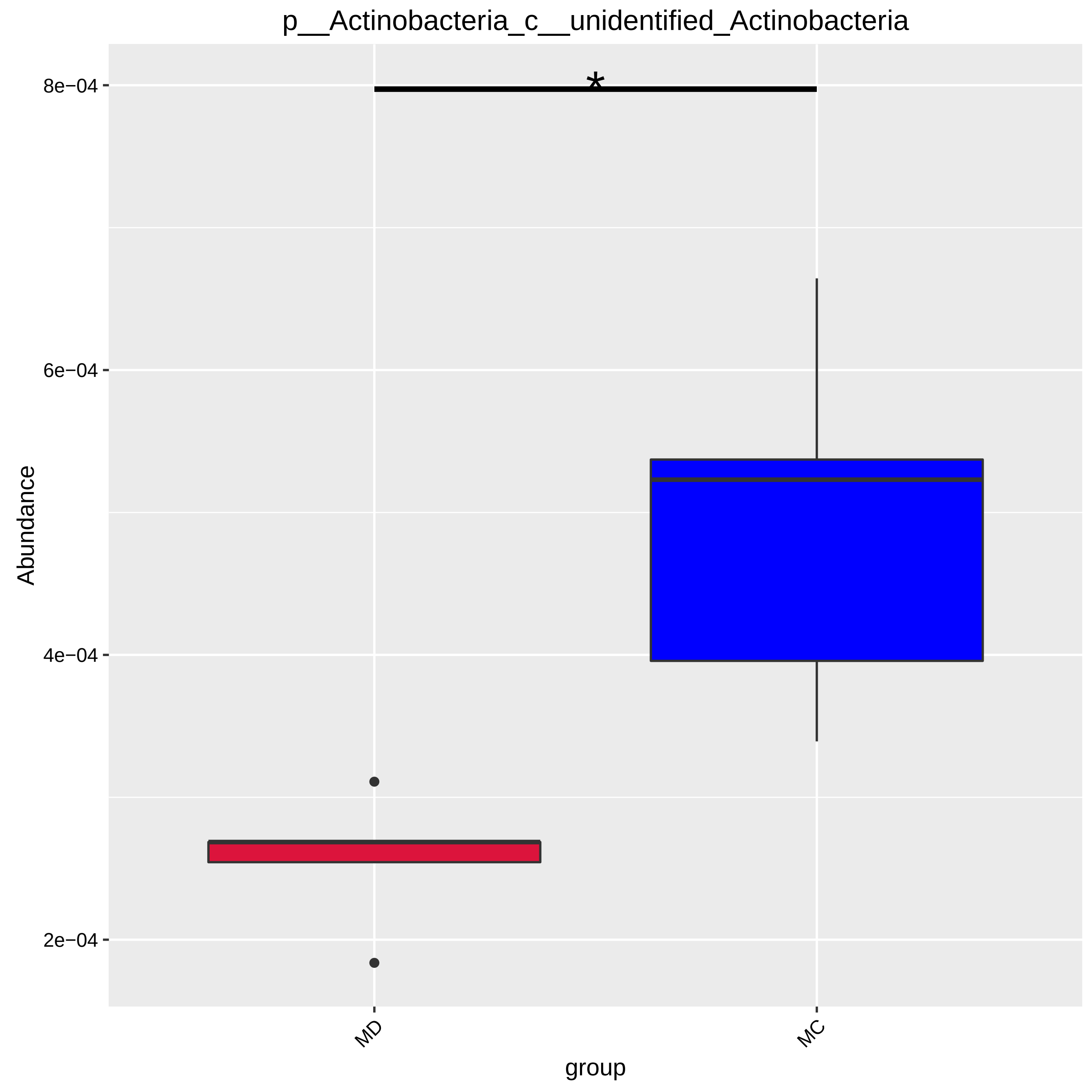
p__Actinobacteria_c__unidentified_Actinobacteria
*
8e−04
6e−04
4e−04
2e−04
Abundance
MD
MC
group
